# Artificial Intelligence in Agro-Food Systems: From Farm to Fork

**DOI:** 10.3390/foods14030411

**Published:** 2025-01-27

**Authors:** Ali Aghababaei, Fatemeh Aghababaei, Marc Pignitter, Milad Hadidi

**Affiliations:** 1 Department of Information Engineering, University of Padova, Via Gradenigo, 6/b, 35131 Padova, Italy; ali.aghababaei@studenti.unipd.it; 2 Aora Health, Calle Via de los Poblados, 17, 28033 Madrid, Spain; aghababaei.afi@gmail.com; 3 Institute of Physiological Chemistry, Faculty of Chemistry, University of Vienna, 1090 Vienna, Austria; marc.pignitter@univie.ac.at

**Keywords:** machine learning, food industry, agriculture sector, computer science, artificial neural networks, algorithms

## Abstract

The current landscape of the food processing industry places a strong emphasis on improving food quality, nutritional value, and processing techniques. This focus arises from consumer demand for products that adhere to high standards of quality, sensory characteristics, and extended shelf life. The emergence of artificial intelligence (AI) and machine learning (ML) technologies is instrumental in addressing the challenges associated with variability in food processing. AI represents a promising interdisciplinary approach for enhancing performance across various sectors of the food industry. Significant advancements have been made to address challenges and facilitate growth within the food sector. This review highlights the applications of AI in agriculture and various sectors of the food industry, including bakery, beverage, dairy, food safety, fruit and vegetable industries, packaging and sorting, and the drying of fresh foods. Various strategies have been implemented across different food sectors to promote advancements in technology. Additionally, this article explores the potential for advancing 3D printing technology to enhance various aspects of the food industry, from manufacturing to service, while also outlining future perspectives.

## 1. Introduction

Artificial intelligence (AI) is a branch of computer science focused on replicating human cognitive processes, including reasoning, learning, and knowledge retention [1]. AI is divided into two categories: strong AI and weak AI. Weak AI is designed to create machines that simulate human behavior and judgments, whereas strong AI posits that machines can truly replicate human cognitive processes and consciousness [2]. Strong AI has not yet been realized, and research in this area is ongoing. AI methods are currently being applied across various fields, including the gaming industry, weather forecasting, heavy and process industries, the food and medical sectors, data mining, stem cell research, and knowledge representation [3,4,5]. AI offers a wide range of algorithms, including reinforcement learning, expert systems (ESs), fuzzy logic (FL), swarm intelligence, the Turing test, cognitive science, artificial neural networks (ANNs), and logic programming. The impressive performance of AI has made it a highly favored tool in industries for decision-making and process estimation, with the goal of reducing costs, enhancing quality, and improving profitability [6].

With the global population projected to increase, food demand is expected to grow by 59 to 98% by 2050. To meet this rising demand, AI is being utilized in various areas, including supply chain management, food sorting, production optimization, quality enhancement, and maintaining industrial hygiene standards [7]. According to Sharma, the food processing and handling industries are projected to experience a compound annual growth rate of approximately 5% at least through 2021 [7]. According to Funes and colleagues, ANNs have been employed as an effective tool for solving complex real-world problems in the food industry [8]. Correa et al. [9] further noted that ANNs simplify the classification and prediction of parameters, contributing to their increased adoption in recent years. Additionally, FL and ANNs have been utilized as controllers to ensure food safety, quality control, yield improvement, and cost reduction in production [10]. AI technologies have also proven valuable in food drying processes, serving as effective tools for process control in this area [11,12,13].

Previous research has demonstrated numerous applications of AI in the food industry, each targeting specific goals. One study focused on the various applications of ANN in food process modeling, though it primarily emphasized the use of ANNs within this particular area [14]. Additionally, the application of AI technologies such as ANNs, FL, and ESs in the food industry has been reviewed, with a particular emphasis on the drying of fresh fruits [11]. A review has examined how food safety remains a primary concern within the food industry, prompting the development of smart packaging systems to meet the needs of the food supply chain. These intelligent packaging systems monitor the condition of food, providing information about its quality throughout storage and transportation. The review on intelligent packaging as a tool to reduce food waste reported approximately 45 recent advancements in optical systems for monitoring freshness. The study focused on meat, fish products, fruits, and vegetables, as these are the most common areas of application [15]. Several studies have been conducted on intelligent packaging, demonstrating that its use plays a crucial role in the food industry. These systems are capable of monitoring the freshness of food products and crops throughout the food supply chain [11,16,17].

Although several studies have explored the use of AI and sensors in the food industry, their scope remains limited. Consequently, a comprehensive review that consolidates all AI applications in the food industry, along with their integration with suitable sensors, would be highly beneficial. To the author’s knowledge, such a review is currently unavailable. This type of resource would provide a valuable one-stop reference for industry professionals, practitioners, and academics, detailing the advantages, limitations, and methodologies of these technologies. Specifically, various types of AI and their recent applications in the food industry will be highlighted, covering several AI techniques such as ESs, FL, ANNs, and ML. In a later section, a critical review is conducted that discusses the primary applications of AI algorithms in the food industry. Following this, the trends in AI applications within the food industry are illustrated.

## 2. Scope and Approach

Food production operates within a complex network of supply chain participants that interact on a global scale. The efficiency of this system is shaped by interconnected international and local factors. Numerous key drivers have been identified as influencing the food supply chain, including (i) the expanding global population and evolving dietary habits, (ii) resource shortages essential for agricultural production, such as fertile land, fresh water, and energy, (iii) climate change, (iv) declining biodiversity, (v) poor governance, and (vi) competing agricultural systems [18].

The current food system is inefficient in addressing these challenges and continues to be excessively wasteful, polluting, and demanding on resources. If these trends continue, it will not be able to provide a healthy, safe, and nutritious diet to a growing global population while remaining within the planet’s ecological boundaries. A shift towards a sustainable food system that can meet these needs necessitates adopting a holistic systems approach [19].

In a systems approach, all elements are in constant interaction, often through feedback loops within the food supply chain. This method is particularly crucial for complex systems like food supply chains, where many interactions occur between various influencing factors through mechanisms that are still not completely understood. Various conceptual approaches to food systems have been created, integrating all activities and their interconnections within the system. These approaches also consider food security alongside socio-economic and environmental indicators [20]. These approaches take a holistic view by broadening the traditional understanding of the food supply chain or farming system to include the interactions within the whole food system and its socio-economic and biophysical environment. Such frameworks enable the development of comprehensive international policies that target food security, nutrition, and global agribusiness. Achieving a truly sustainable food system requires aligning the digital strategy vision with the objectives of the Green Deal. In this framework, we outline our vision for a systems approach to food production, highlighting how digital technologies and AI can assist in overcoming the challenges associated with it.

## 3. Artificial Intelligence: State-of-the-Art Techniques

DL and machine learning (ML) are two highly utilized AI techniques across individual, business, and governmental sectors, leveraging data-driven models to enable accurate predictions. In the food industry, recent developments in ML aim to manage the complexities and uncertainties of vast information [21]. Core subfields within AI, such as ANNs, robotics, ESs, computer vision systems (CVSs), natural language processing (NLP), and ML, each contribute distinct capabilities. NLP aids in interpreting human language, CVSs facilitate digital-to-analog conversion (e.g., speech recognition and video analysis), and ESs replicate human-like decision-making. The three cognitive functions underlying AI are learning (data acquisition and algorithm development for deriving actionable information), reasoning, and self-correction [22].

Advances in AI have recently expanded its use within the agro-food sector, where it plays a critical role in identifying models, generating services, and supporting decision-making. AI contributes substantially to agricultural productivity by providing accurate, predictive insights for optimizing resource usage [23]. Specifically, AI algorithms classify patterns, predict potential issues, and aid in managing agricultural challenges, such as pest identification and treatment recommendations, irrigation, and water management via smart irrigation systems. Remote sensing and sensor technology assess biotic and abiotic factors, enhancing agricultural and livestock management practices [22].

The data acquisition process gathers real-world information from sources such as sensors, historical records, and customer feedback. This is followed by data pre-processing—cleaning, normalizing, and standardizing the data to ensure its consistency and quality. Next, pertinent features are extracted from the data through feature engineering to develop an AI model capable of accurately predicting output quality. The pre-processed data then undergo model training, allowing for accurate product quality predictions. Throughout this process, human oversight plays a vital role in tasks such as data annotation, model validation, interpretability, and ethical compliance, ensuring that the AI model functions effectively and adheres to quality standards and regulations [22].

### 3.1. Machine Learning

**Figure 1:**
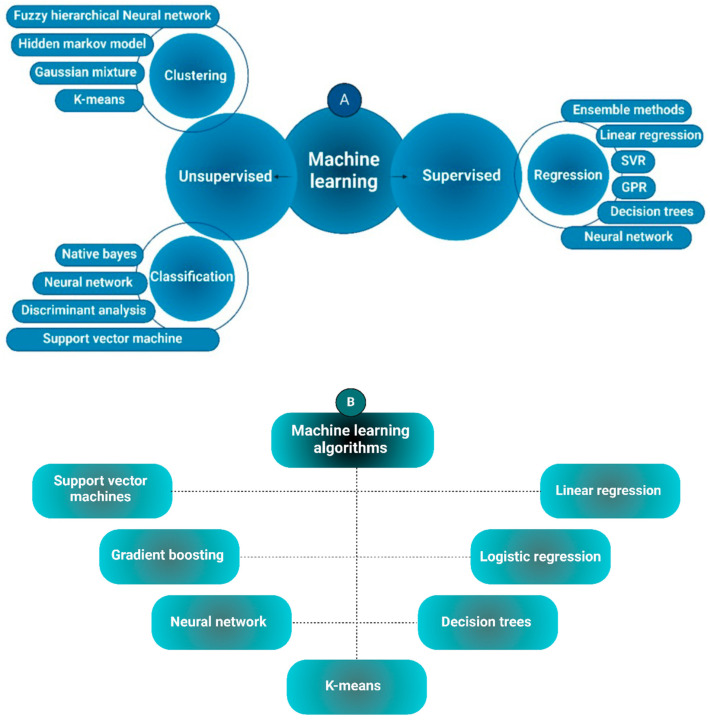
(**A**) classification of ML and (**B**) ML interpretation in the food business using different algorithms.

ML is a pivotal AI field driving the advancement of creative and productive work. As illustrated in Figure 1A, ML can be categorized into two primary types—supervised and unsupervised learning—each encompassing various methodologies applicable across diverse domains, including food processing, as depicted in Figure 1B. The principal ML tasks are supervised and unsupervised learning [24].

In supervised learning, the process requires labeled data and oversight. Here, computers are trained on a “labeled” dataset, allowing them to accurately map input to output variables. After training, the model is able to make predictions based on these mappings. Applications of supervised learning span risk assessment, spam filtering, and fraud detection. Conversely, unsupervised learning enables computers to analyze data independently using unlabeled datasets [25]. This method involves grouping or categorizing data based on patterns, similarities, or differences, with the aim of discovering hidden structures within the dataset. ML leverages mathematical and statistical methods to draw insights and make decisions from data. Broadly, ML techniques fall into symbolic and sub-symbolic approaches. In supervised learning, for instance, the goal is to create a predictive model that maps input variables to a specified output variable using labeled data [26]. Among the algorithms frequently utilized in supervised learning are decision trees (DTs), Bayesian networks (BNs), and regression analysis.

In contrast, unsupervised learning operates on unlabeled data to uncover hidden patterns, primarily applied in tasks like dimensionality reduction and exploratory data analysis. This approach combines training and test data, allowing the learner to engage with its environment to gather insights. Through exploratory actions, rather than relying solely on pre-existing data, the learner enhances its understanding by experimenting with new, untested actions [27].

#### 3.1.1. Deep Learning

DL is a subset of ML that involves training ANNs with multiple layers to model complex patterns in data. This approach has been particularly successful in tasks such as image and speech recognition, natural language processing, and game playing [28]. The development of DL has been significantly influenced by researchers like Yann LeCun, Yoshua Bengio, and Geoffrey Hinton, who have contributed foundational work in this field. For instance, LeCun’s work on convolutional networks has been pivotal in advancing image recognition technologies. The success of deep learning is attributed to its ability to automatically learn hierarchical representations from raw data, enabling the modeling of DL to form a cornerstone of modern AI research.

**Figure 2:**
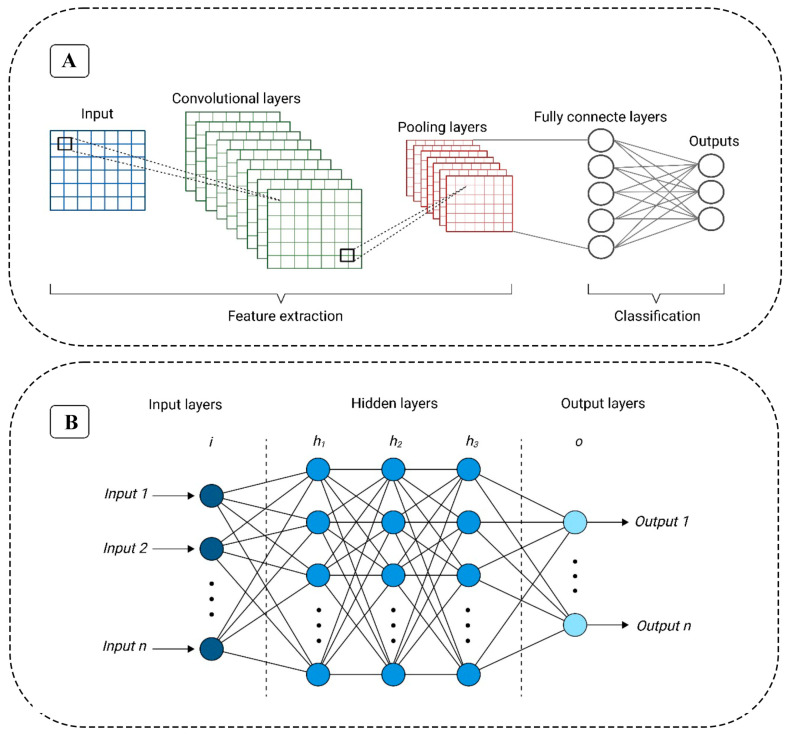
A schematic representation of (**A**) a CNN and (**B**) an ANN.

DL recently experienced significant advancements in the detection of food adulteration and defects. This progress began in the late 1990s with the development of convolutional neural networks (CNNs), which have become one of the most powerful methods for learning intricate features of digital data in tasks related to classification and regression [29]. Unlike traditional neural networks, CNNs employ multiple convolutional layers, as illustrated in the standard CNN architecture for food analysis and detection in Figure 2A. These convolutional layers utilize filters to extract features from input images, with specific parameters—such as padding, stride, kernel size, and activation functions—tailored to the task at hand and optimized for effectiveness.

At the network’s output end, fully connected layers act as classifiers, utilizing a densely connected block to generate the final prediction. Various CNN architectures have been developed for image classification in food and agricultural applications, including LeNet, AlexNet, VGG, GoogLeNet, ResNet, and Inception. CNNs have proven highly effective in identifying food adulteration and evaluating the quality of agricultural products. However, GoogLeNet, ResNet, and Inception do not specifically detail their performance in food and agriculture applications [30].

#### 3.1.2. Artificial Neural Network

An ANN represents a sophisticated component of AI with substantial potential for application within the food industry. Particularly in unsupervised learning, ANNs leverage unlabeled datasets, functioning without predefined input and output variables. Techniques such as ANNs, clustering, genetic algorithms, and DL play a vital role, with ANNs being the most prominent approach in evaluating food quality within AI-based applications. Structurally, an ANN is a network of nodes that can perform either linear or nonlinear processing tasks [31]. Theoretically, electrical impulses pass through synaptic connections at each node within the interconnected network, moving through neuron-like structures with the aid of axons [32]. ANNs operate on principles similar to the human brain, triggering internal operations rather than conventional computational processes.

A standard ANN architecture comprises three main layers: input, hidden, and output (as illustrated in Figure 2B). The network’s architecture incorporates activation functions, with data processed through feed-forward or feedback mechanisms. The input layer initially receives raw data, which then flows to the hidden layer for processing before reaching the output layer through a sequence of interconnected nodes. One major advantage of ANN layers lies in their ability to facilitate parallel reasoning, making neural networks highly effective for prediction. Similar to a human brain, an ANN can learn and store synaptic weights, representing connections between neurons [22].

According to Sukhadia and Chaudhari [33], ANN structures are tailored to support specific applications, such as pattern recognition and data classification. Furthermore, Gonzalez-Fernandez et al. [34] emphasize that ANNs are adaptable, versatile, and suitable for various scenarios. Although some modifications may be necessary, ANNs are highly flexible and can simulate a wide range of nonlinear systems. Nonlinear regression is among the network’s most distinct capabilities. The multilayer perceptron (MLP) model, frequently used for pattern recognition and predictive tasks, processes input data in its hidden layer to form internal representations. Throughout training, the network’s parameters—weights and biases—interact in complex ways to develop these representations. While the opaque, “black-box” nature of ANNs often challenges precise interpretation, tools such as feature analysis, visualization, and weight interpretation offer insights into the patterns recognized by neurons within the hidden layer [22].

### 3.2. Computer Vision System

**Figure 3:**
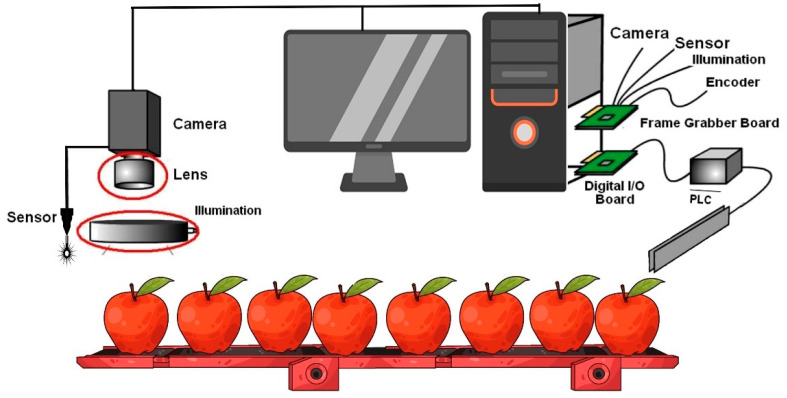
Schematic representation of CVS.

Lukinac et al. [35] describe a series of procedures in CVSs, including image processing, digitalization, analysis, and capture (Figure 3). This method relies on the visible spectrum interacting with a reflective or absorbent material, capturing images through image analysis. During this process, photons are captured by camera lenses and then converted into electrical signals by an image sensor. Digitization involves transforming these images into a numerical format for further analysis. They observed that a CVS can quantify external attributes in digital images, facilitating automated quality control of products [35]. As AI continues to progress, integrating ML, ESs, FL, and CVSs offers substantial opportunities for innovation across multiple fields. For complex, multi-layered production and consumption networks, optimizing AI models is essential to increase efficiency, adaptability, and resilience. Streamlined AI models can reduce resource waste, lower production costs, and enhance productivity. Effective resource allocation is crucial in this optimization, with algorithms designed to ensure resources are distributed across network nodes, minimizing shortages or excess.

AI-driven supply chain management also focuses on precise demand forecasting, inventory optimization, and disruption identification, all contributing to uninterrupted operations. Additionally, AI can integrate risk mitigation measures, identifying and addressing potential risks, such as supply chain disturbances or market volatility. AI models continuously adapt to shifting conditions, enhancing resilience and maintaining performance in dynamic environments. Through these optimization strategies, AI contributes to greater efficiency, flexibility, and reliability within complex networks, helping to meet the demands of rapidly changing operational landscapes [22].

### 3.3. Fuzzy Logic

FL effectively simulates the complex decision-making processes humans use when faced with ambiguous or uncertain information. It is recognized as a valuable approach for managing classification challenges involving incomplete data. However, FL requires enhancement in handling recovery processes, especially under constraints in high-dimensional or large datasets. Fuzzy systems facilitate simplified algorithmic formulations and linguistic variables to represent the behavior of complex systems qualitatively rather than relying on quantitative or mathematical models. FL has been instrumental in developing control systems for sophisticated food and beverage manufacturing processes.

**Figure 4:**
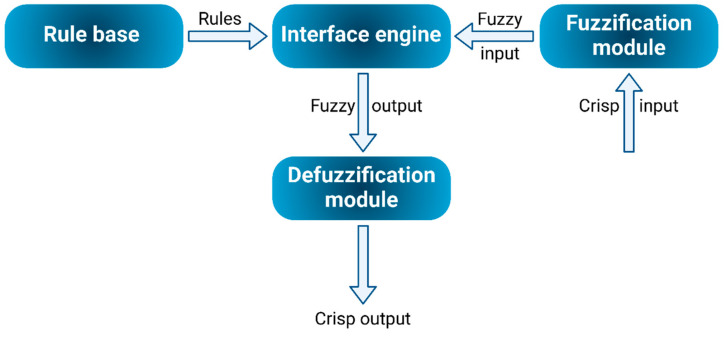
The main four parts of FL.

A typical FL architecture, as illustrated in Figure 4, comprises four essential components: the rule base, fuzzification, inference engine, and defuzzification. According to fuzzy set theory, an element is part of a fuzzy set if it is associated with a real number between 0 and 1 [36]. FL models involve various stages, including fuzzification, inference, and defuzzification [37]. Fuzzification transforms a crisp input value into a degree of membership within a fuzzy set, generally represented on a scale from 0 to 1. The most commonly used membership functions include triangular, Z-shaped, S-shaped, trapezoidal, and Gaussian forms, though numerous other types exist [38].

The inference system is where fuzzy rules are applied to translate fuzzy inputs into corresponding outputs, leading to the final defuzzification stage [39]. Defuzzification methods vary, with common techniques including the mean of maximum, center of maximum, center of gravity, centroid of area, smallest of maximum, and largest of maximum. Villasenor-Aguilar et al. [40] applied FL to predict total soluble solids and assess bell pepper maturity stages, reaching an 88% classification precision for the four stages of maturity. Additionally, Pakyürek et al. [41] utilized FL for quality grading across three pineapple varieties.

These studies highlight FL’s robustness as a control mechanism, particularly in handling intricate processes essential for assessing food and agricultural product quality.

### 3.4. Expert System

**Figure 5:**
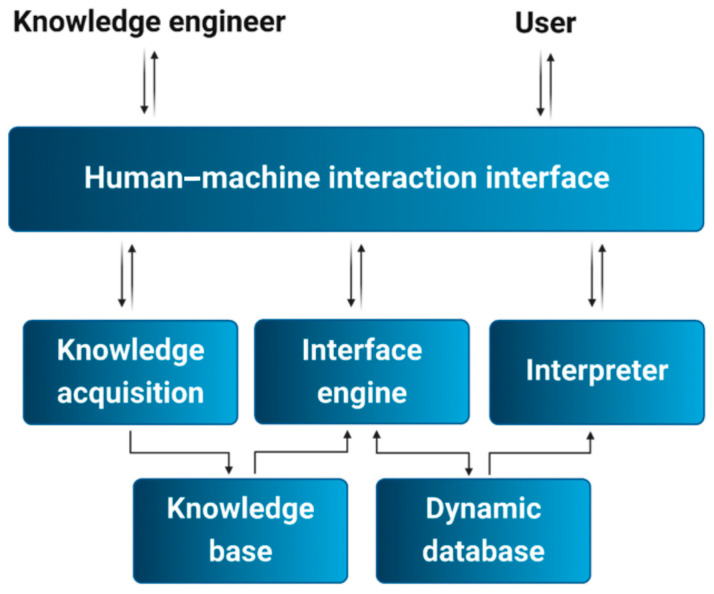
Schematic representation of ES.

An ES, a subset of AI, leverages specialized knowledge and reasoning capabilities to tackle complex problems through structured knowledge representation. Typically, an ES comprises an interpreter, an inference engine, a dynamic database, a human–machine interface, and a knowledge acquisition module (Figure 5). The problem-solving component of an ES is designed to mimic expert-level reasoning by processing knowledge and information systematically. Recently, ESs have been implemented on standard devices with optimized variables and multiple predictive factors, enhancing their performance. By selecting features based on color, texture, and geometry, ESs have achieved 100% accuracy in classifying fruit ripeness [22].

Duong et al. [42] created an ES utilizing EfficientNet and MixNet deep neural network (DNN) architectures to identify fruits. These architectures significantly improved classification accuracy by up to 95% compared to a widely recognized baseline. To ensure ESs are commercially viable for quality control in food and agriculture, rigorous development processes are essential. Evaluating ESs involves assessing their processing speed, monitoring capabilities, and effectiveness in automated detection tasks. As input features are readily available, the system’s accuracy and operational efficiency are key metrics for successful implementation.

## 4. Role of AI in Agriculture

The integration of ML algorithms is playing an increasingly critical role across the four key stages of the agricultural supply chain: preproduction, production, processing, and distribution [43]. In the preproduction phase, ML technologies are particularly valuable for forecasting crop yields, assessing soil characteristics, and determining irrigation needs. During the production stage, ML can be utilized for detecting crop diseases and predicting weather patterns, which are essential for optimizing agricultural output. In the third phase, which focuses on processing, ML methods are primarily employed to optimize production planning, ensuring both high-quality and safe products. Additionally, ML algorithms play a crucial role in the distribution phase, particularly in areas such as storage management, transportation logistics, and consumer behavior analysis. The preproduction phase, which is the starting point of the agricultural supply chain, involves the use of ML for forecasting crop yields, evaluating soil characteristics, and determining irrigation requirements. Numerous studies highlight the significance of crop yield prediction in enhancing plant management practices. By incorporating input data such as equipment needs, nutrients, and fertilizers, ML-based predictive models are being developed as part of precision agriculture tools. These models assist stakeholders and farmers in making informed decisions regarding crop yield forecasting, thereby advancing smart farming techniques. Recently, a variety of ML algorithms, including BN, regression models, DTs, clustering methods, DL, and ANNs, have been employed to predict crop yields [44,45,46]. In the field of soil property prediction, various ML algorithms have been applied to analyze and understand soil characteristics. For instance, Morellos et al. [47] employed the least squares support vector machine (SVM) method to study a dataset of 140 soil samples. In another study, Kumar et al. [48] introduced an innovative approach called the crop selection method to address challenges in crop selection and enhance the overall net yield of crops throughout the growing season. Furthermore, Ben Ayed et al. [44] conducted an analysis of 18 different table olive cultivars from around the world. By examining morphological, biological, and physicochemical traits, alongside utilizing a BN, they explored how these factors impact tolerance, productivity, and oil content in the cultivars. The findings revealed that crop tolerance had a significant impact on oil content. Additionally, irrigation management plays a crucial role in the preproduction phase, as it greatly affects both the quality and quantity of the crops. To develop more efficient irrigation systems—determining the optimal timing, location, and amount of irrigation—researchers have utilized input data such as soil moisture levels, precipitation, evaporation rates, and weather forecasts. These data are then fed into ML models for simulation and optimization, improving decision-making in irrigation practices [49]. Arvind et al. [50] demonstrated the effectiveness of integrating ML algorithms with technologies like sensors, Zigbee, and Arduino microcontrollers for drought prediction and mitigation. Similarly, Cruz et al. [51] applied an ANN using feed-forward and back-propagation methods to optimize water resource management within smart farming systems. In a more recent study, Choudhary et al. [52] utilized partial least squares regression alongside other regression algorithms as part of an AI toolkit, combining them with sensors for data collection and IoT hardware to boost efficiency and economic viability. The production phase represents the second stage of the agricultural supply chain, where various critical factors influence crop production. Key parameters include weather forecasts, such as sunlight, rainfall, and humidity, as well as crop protection strategies against both biotic stressors (such as pathogens and weeds) and abiotic stressors (including nutrient and water deficiencies). Additionally, crop quality management and harvesting practices play essential roles in ensuring successful production outcomes. A variety of ML algorithms are employed to create effective models across different aspects of agriculture. For weather prediction, algorithms such as ANNs, DL, DTs, ensemble learning, and instance-based learning are commonly used [53]. In crop protection, clustering and regression methods are applied [54], while ANNs, DTs, DL [55], and instance-based learning [56] are utilized for weed detection. Crop quality management typically involves clustering and regression algorithms [57], and for harvesting, DNNs, along with data mining techniques like K-means clustering, K-nearest neighbor, ANNs, and SVM, are leveraged [58].

In the final horticultural phase, which occurs during the harvest stage after the crops have ripened, ML algorithms are also used to predict changes in the color of fruits or crops. Numerous research teams have employed ML techniques to predict the stages of fruit ripening and maturity. For instance, Gao et al. [59] attained a 98.6% accuracy in classification when they utilized hyperspectral datasets along with the AlexNet-CNN model to categorize strawberries into early-ripening and fully ripe stages. The processing cluster represents the third phase in the agricultural supply chain. Various processing methods are employed for agricultural products, including techniques like smoking, heating, cooking, cooling, milling, and drying. Choosing the optimal combination of parameters during this processing phase guarantees both high-quality and high-quantity food production, while also minimizing the overuse of resources. To accomplish this, many food industries have adopted modern food processing technologies, integrating software algorithms powered by ML. Some of the commonly utilized ML algorithms include genetic algorithms, ANNs, clustering techniques, and BNs [60]. Arora and Mangipudi [61] introduced models based on SVM classifiers and ANNs to detect nitrosamine in red meat samples. Their predictive modeling results indicated that the DL model achieved the highest accuracy during testing. Farah et al. [62] combined differential scanning calorimetry with ML techniques such as gradient boosting machine (GBM), random forest (RF), and MLP to analyze milk characteristics, verify its authenticity, and detect fraudulent activity. The best outcomes were obtained using GBM and MLP, which were able to accurately classify 100% of the adulterated samples. The distribution cluster represents the final stage of the agricultural supply chain, acting as the connection between food production, processing, and the consumer. ML algorithms can be utilized in various areas, including storage, transportation, consumer analytics, and inventory management. In the storage and transportation phases, popular algorithms like genetic algorithms, clustering, and regression techniques are frequently employed. These predictive techniques are developed to improve food quality preservation, guarantee product safety, and minimize damage by monitoring the product across the entire supply chain [44]. In the consumer analytics phase, ML techniques such as DL and ANNs are applied in food retailing to predict consumer demand, preferences, and purchasing patterns. For inventory management, genetic algorithms in ML are utilized to forecast daily demand and assist in avoiding inventory problems [63]. There are numerous examples of AI-driven technologies in the agri-food industry, including mechatronics and robotics [64], drones [64], geographic information systems (GIS) [65], blockchain (BC) [66], and satellite-based guidance systems [64]. Miranda et al. [67] highlighted these technologies as sensing, smart, and sustainable, offering systematic processes characterized by connectivity, automation, precision, monitoring, and digitization [68,69,70]. In agriculture, robotics, mechatronics, and smart mechanization are designed to lessen manual labor and optimize resource use by employing highly autonomous and intelligent machinery [64]. The transition from horses to tractors, robots, and intelligent vehicles marks a revolutionary period for agriculture and the food industry. This shift has brought agriculture from basic methods to highly efficient practices, thanks to mechanization, innovative technologies, computerized analysis, and decision-making systems, all of which enhance farming operations and boost crop productivity [64]. Innovative machines, commonly referred to as “agribots”, are now widely utilized in agriculture for various tasks such as soil preparation, seed planting, pest and weed control, irrigation, fertilization, and even harvesting grains and fruits, significantly reducing labor and energy costs [71]. Agricultural drones play a key role in overall crop management, beginning with soil treatment using herbicides, progressing through sowing and plant treatment with pesticides, conducting physiological monitoring and observation, and culminating in determining the optimal harvest time [32,72,73,74]. Today’s agricultural drones can not only deliver fertilizers, pesticides, water, and herbicides but also capture images, record videos, and produce real-time maps of fields and crops. This technology aids farmers in making informed management decisions [75,76].

Farmers now employ drones for livestock monitoring, allowing them to track health conditions such as illnesses, injuries, and pregnancies with greater precision. The market for agricultural robots and drones is anticipated to grow significantly, with estimates projecting it to reach USD 23 billion by 2028 [77]. Geospatial technology, which utilizes satellite data, enables the application of GIS across various agricultural domains. These applications encompass crop management, irrigation control, yield forecasting, disease and weed management, farm automation, livestock monitoring, vegetation mapping, and predicting land degradation and erosion. GIS is especially effective for precision agriculture, real-time monitoring, and improving situational awareness, playing a vital role in meeting the increasing global food demand. Additionally, BC technology is increasingly used to meet consumer concerns about food origin, quality, and, most importantly, safety [78].

## 5. Role of AI in Food Industry

AI has been applied across various fields, including modeling, classification, and data analysis. Example applications include numerical processing, control systems, communication technologies, interpreting data from pyrolysis, gas chromatography, mass spectrometry, and HPLC. AI is also used in pattern recognition of RNA, protein, and DNA structures, predicting microbial growth, biomass, and food shelf life and detecting pathogens [79]. In the food industry, ANNs, FL, and genetic algorithm techniques have been employed to enhance performance in handling and engineering processes. AI has been applied in food science and processing for tasks such as sorting, quality control, and wine analysis. Systems like Clean-In-Place (CIP) and Clean-Out-of-Place are crucial for maintaining hygiene and ensuring high product standards in the food industry, often utilizing AI for optimization, known as self-optimizing-CIP. However, with the growing global population, food production remains insufficient due to shrinking agricultural lands, climate change, and increasing pollution levels [80]. These factors are negatively impacting food production, the environment, nutrient availability, and human health. Billions of people worldwide continue to suffer from nutrient deficiencies. Comprehensive strategies can be employed to create incentives that drive progress and improve food security outcomes [80].

In response to public demand, farmers have adopted new harvesting techniques that leverage AI to boost yields [81]. The advancement of horticulture and related technologies has led to numerous new studies. AI in agriculture involves data analysis, decision-making, and the application of machine power for the early detection of crop diseases, providing livestock with optimal nutrition, and enhancing agricultural inputs and profits based on supply and demand dynamics [79]. Some technologies, such as pest control management and pesticide data, help farmers increase their yields. A promising solution to these challenges is the BN. This method is user-friendly and does not require advanced software skills, making it accessible even to beginners. The Bayesian approach allows for simulations by integrating prior knowledge into data analysis to produce predictive insights. Unlike traditional frequentist methods, it does not rely on null hypothesis testing. Instead, it helps calculate mutual information, which reflects the probability between data sources [80]. By applying these techniques within a BN, the interactions between theoretical factors in the Global Food Security Index can be analyzed. These Bayesian models are especially applicable to global food safety, providing intuitive and user-friendly visualizations. AI is essential in the food industry, contributing not only to quality control and food security but also to areas like sanitation, manufacturing, and packaging.

**Figure 6:**
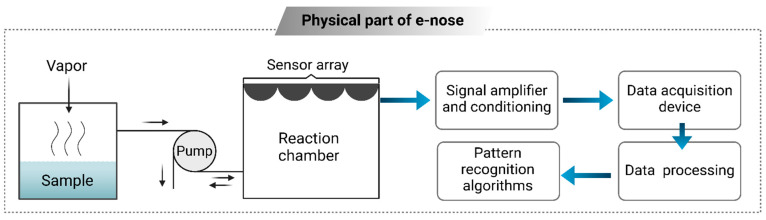
A schematic representation of e-nose system.

As a branch of AI, the CVS merges techniques from image processing and pattern recognition. This approach is non-destructive, allowing for the analysis and extraction of features from images, which aids in developing classification patterns [82]. The CVS is acknowledged as a valuable tool for extracting external feature measurements, including color, shape, size, and defects. Typically, this system consists of a digital camera, a lighting setup, and software designed to process images and perform analyses [83]. The system can be categorized into two types: 2D and 3D versions. Its application spans multiple areas within the food industry, including predicting color attributes in pork loin [84], assessing the ripeness of apples [82], determining the roasting level of coffee [85], identifying defects in pork [86], and evaluating the quality of table grapes [87]. Integrating the CVS with the electronic nose (e-nose) system and soft computing techniques is recognized as a significant and valuable asset in the food industry (Figure 6). This combination provides notable benefits, including the ability to achieve accurate predictions rapidly [2]. Table 1 illustrates how the integration of the CVS and soft computing has been applied within the food industry.

### 5.1. AI in 3D Printing

3D printing refers to a collection of additive manufacturing processes that originated in the late 1980s, transitioning from prototyping tools to a disruptive technology ecosystem. While the methods may vary, they share common applications, primarily involving the construction of material layers. For instance, metal powder is melted using lasers, or liquid polymers are solidified with ultraviolet light to create the desired structure. This technology enables the creation of complex shapes and allows for the generation of digital models, facilitating decentralized production and customization of parts [101]. 3D printers function similarly to robots. The increase in robotics manufacturing is driven by the ease of electronics development, the accessibility of powerful cloud computing, and the availability of high-quality sensors. In the past, robots were prohibitively expensive and primarily utilized in large-scale industries such as automotive manufacturing, where companies would typically invest at least USD 1,000,000 to incorporate them into production lines [102]. 3D printing and robotics are significantly transforming the confectionery industry, offering faster and more efficient operations [79].

The process begins with a digital file created in a computer-aided design software environment, where every aspect of the model can be precisely defined, modified, and optimized using 3D design tools. These advancements have captured the attention of companies seeking streamlined production methods [103]. Once the digital model is complete, it is saved as a .stl file and then transferred to the 3D printing software interface. Many 3D printing programs are open source and free to use. The software slices the object into layers and generates individual commands. Key parameters such as speed, temperature, height, and thickness, often controlled by robotic arms, are configured within the 3D printing software. The completed design and its features are then exported in a format compatible with the 3D printer for production [104].

A state-of-the-art 3D production system, equipped with a culinary innovation center utilizing 3D printing technology, has been introduced to allow chefs in the food industry to experiment with the ChefJetTM Pro professional food printer (3D systems, California, USA). In partnership with Hershey, a prototype 3D chocolate printer called CocoaJet (Cocoapress, Florida, USA) was unveiled in 2015. In Australia, TM Retail Food Group began implementing 3D chocolate printing for personalized cake messages within their Michel’s Patisserie franchise, with plans to roll it out nationwide by 2018 [105,106]. After making modifications to commercial printers, the equipment successfully obtained a “food grade” certification from the Federal Agency for the Safety of the Food Chain.

The use of software is a critical factor in designing, slicing, and optimizing the model. Slicing software (ver 1.0) serves as a bridge, enabling the planning and assessment of the layers between the 3D model and the 3D printer. This software converts the digital model into a physical form by translating .stl files into g-code for 3D printing. Setting up the software is relatively simple, requiring only a few adjustments. Key factors such as nozzle temperature, printing speed, layer thickness, and platform temperature are essential to optimize the printing process, along with features for support design and model repair [107].

### 5.2. AI in the Dairy Sector

Milk is transported from various production centers to processing plants via milk trucks. Once at the plant, the milk undergoes pasteurization, a process that eliminates bacteria and extends the milk’s shelf life. The pasteurized milk is then used to produce a variety of dairy products. For optimal quality, milk should have a pH value of 6.7. If the pH deviates from this level, the milk spoils rapidly. Refrigeration helps prevent spoilage, and this process is typically carried out at milk stations to preserve the product until it reaches the collection point. To address this issue more effectively, a wireless sensor network integrated with AI technology is needed to monitor and maintain the quality of the milk throughout the supply chain [108]. AI also plays a significant role in automating the milking process in the dairy industry. Traditionally, manual milking is time-consuming, but this can be minimized with the use of Automated Milking Systems or milking robots. Although this technology was introduced in the US in 2000, it was first developed in Europe in 1992 [109]. By 2002, nearly 1754 milking robots were in use, a number that grew to 8190 within five years, and by 2010, the total had risen to 16,000 [109].

In 2010, Germany and France accounted for 30% of the robots in operation [110]. According to experts, the robotic milking market is expected to support 28,600 robots annually. Cows are trained for the automated milking process and equipped with electronic tags, enabling the robot to recognize each cow and dispense feed. The robot attaches milking cups to the teats, initiating the milking procedure. As each quarter completes milking, the cups are automatically removed, and a disinfectant is applied before the cow leaves the milking station [109]. Robots also conduct disease detection tests on milk using technologies such as laser scanners, ultrasound, and the Optical Guidance System. When no signs of disease are detected, the milk is directed for cooling [110]. These robots utilize a CVS for navigation and sensing, while ML, a critical aspect of AI, allows them to learn from human interactions [109]. Programmed CIP systems handle the cleaning process with two distinct programs: (a) a CIP program for rotary cleaning, which includes equipment such as heating surfaces and pasteurizers, and (b) a CIP program for rotational cleaning, which includes tanks used for storing purified milk [79].

With AI integration, operators can select specific steps and run programs based on signals from the system, allowing them to monitor temperature levels for different liquids in various tanks. Unified CIP systems are commonly used in large milk sectors for separation processes. In the dairy industry, robots are employed for cheese packaging, cutting, and curd slicing according to customer-specified shapes and sizes. Special grippers enable the robots to pick up cheese blocks and place them on conveyors for further processing [109].

### 5.3. AI in Food Processing Strategies

**Figure 7:**
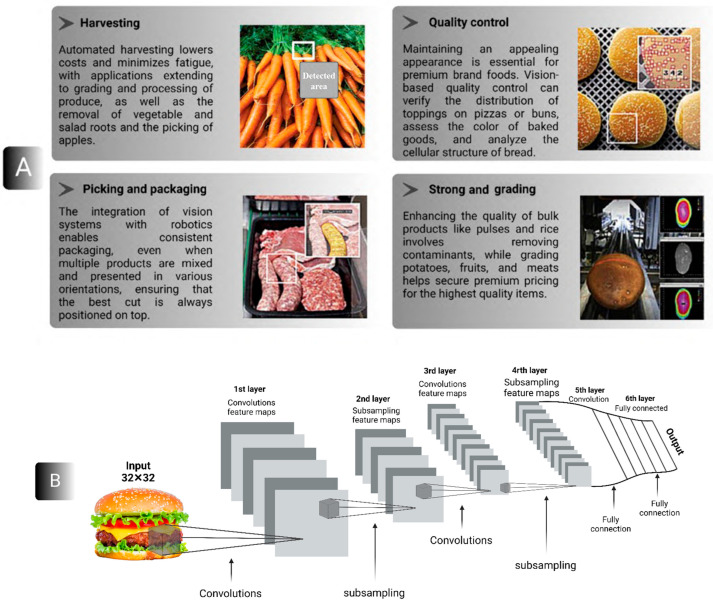
AI and computer vision applications in the food industry: (**A**) harvesting, quality control, picking, and sorting utilizing vision algorithms; (**B**) food classification with LeNet-DL architecture.

The vast amount of data surrounding food and edible products have driven researchers to investigate this field through the application of AI. By 2015, computers had advanced to the point where they could identify food from images. By early 2016, MIT’s AI had the capability to predict ingredients and assess nutritional values based on the food presented to it. This technology quickly reached the public, becoming available as a mobile application within a few months [111]. AI technologies assist the food industry in efficiently promoting products to the market through global food trend strategies and planning [112]. Food processing becomes more adaptable with machines capable of handling tasks ranging from simple distinctions, like identifying apples versus oranges, to more complex tasks, such as differentiating between low-saturated and high-unsaturated fats. Figure 7A illustrates food monitoring techniques used at “Stemmer Imaging (Puchheim, Germany” for various applications, while Figure 7B demonstrates the application of DL in food classification using the LeNet architecture.

### 5.4. AI in Food Safety

**Figure 8:**
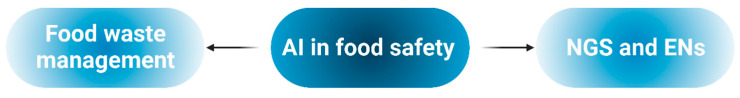
AI in food safety.

The sterile characteristics of robots make them highly suitable for use in food processing industries, significantly contributing to the reduction in foodborne diseases. This aspect is particularly important in light of the stricter hygiene requirements outlined in the Food Safety Modernization Act, which applies to the entire supply chain. The primary concern is that products like cereals, spices, and other shelf-stable foods, which do not require refrigeration, are particularly vulnerable to contamination. While these foods were once considered less prone to contamination, the situation has dramatically changed in recent times. AI-based systems offer a promising solution to these challenges, as they are not susceptible to transmitting illnesses in the same way humans can. Furthermore, maintaining AI-driven systems is straightforward and efficient, making them an ideal choice for ensuring hygiene and reducing the risk of contamination in food processing environments [113]. A report by Technavio indicates that the adoption of robots in food processing industries is expected to increase by 30%, aligning with government regulations. Additionally, several innovative applications of AI in food safety are emerging and are expected to gain widespread recognition in the near future. These advancements aim to significantly reduce the occurrence of foodborne diseases. Figure 8 illustrates the role of AI in enhancing food safety [114].

#### 5.4.1. Next-Generation Sequencing and Electric Noses

Two of the most promising innovations in the food industry are next-generation sequencing (NGS) and ENs. NGS is rapidly replacing traditional DNA-based methods in food safety applications. The integration of AI-powered automated systems and workflows has significantly accelerated data collection and laboratory testing, making these processes faster and more accurate than ever before. NGS is highly effective at quickly detecting potential hazards, enabling the prevention of widespread infections before they impact a large number of individuals. ENs function as substitutes for human olfactory senses in manufacturing environments. These devices are equipped with sensors that can accurately detect a wide range of odors. The sensors capture the surrounding smells, and the collected data are transmitted to a centralized data center, where ML algorithms analyze the information [113,115]. Based on the analysis of the ML-based system, an alert is sent to the production units when necessary. As a result, ENs have the potential to become a key technology in the future of food safety.

#### 5.4.2. Food Waste Management

According to a report from the U.S. Department of Agriculture, food waste in the United States is estimated to account for 30 to 40% of the total food supply. This estimate, derived from the USDA’s Economic Research Service, indicates that 31% of food loss occurs at the retail and consumer levels, which equates to approximately 133 billion pounds and USD 161 billion worth of food in 2010. This level of waste has significant and widespread social implications [114]. Moreover, AI has the potential to address food waste issues and create significant opportunities by reducing waste levels by 2030. These impressive outcomes can be achieved through the implementation of additional regenerative agricultural practices [116].

This indicates that resources are not being utilized optimally. Traditional farming methods can be replaced by more advanced, intelligent farming techniques. These methods involve the use of various sensors to gather data which are then processed using ML algorithms to make informed decisions. By adopting these approaches, farmers can make faster and more accurate decisions. Below are some suggested strategies to reduce food waste through the use of AI:

- While some studies focus on assessing the ripeness of fruits, others highlight the role of beneficial microorganisms in promoting the growth of fruits and vegetables without the need for artificial fertilizers;
- Producers can eliminate the need for soil testing by leveraging the benefits of AI, leading to significant cost savings;
- Farm-based food supply chain management employs CVSs to monitor and analyze each step of the process, leading to a significant reduction in food waste;
- AI-driven food tracking systems will allow for the sale of food before it becomes waste. This approach facilitates greater connectivity between farmers and consumers, promoting more efficient food distribution.

The implementation of such concepts cannot be achieved by a single organization or entity alone. Transforming the entire food industry is essential, requiring a collaborative effort from a network of stakeholders. A unified approach is necessary to develop an efficient system that can have a significant global impact.

### 5.5. AI in Beverage and Soft Drinks

Drinks can be divided into three main types: (i) alcoholic beverages, (ii) non-alcoholic beverages, and (iii) hot beverages. Alcoholic drinks consist of beer, wine, and spirits, while non-alcoholic options include milk, soda water, juices, and soft drinks like Thumbs Up, Miranda, and Coca-Cola. Hot beverages cover items such as coffee, tea, and hot chocolate [117]. The process of beer fermentation starts by introducing yeast to the aerated wort. The yeast feeds on the nutrients in the wort, initiating its growth and simultaneously generating alcohol and other metabolites. Fermentation proceeds until the sugar levels in the wort drop to a specified concentration, leading to increased alcohol production as the wort becomes lighter. Crucial factors that must be controlled during this process are oxygen levels, temperature, and the yeast pitching rate. Other factors that impact fermentation include wort composition and yeast condition. Inconsistencies in yeast and wort can be mitigated by adjusting the pitching rate or slightly increasing oxygen levels and temperature when yeast viability is reduced. In traditional brewing, continuous monitoring is challenging, but with the use of AI and its programmed tools, the brewing process can now be effectively monitored and controlled in real-time [118].

#### 5.5.1. AI in Beer and Wine Processing

AI approaches such as CVSs, FL, neural networks, and hybrid intelligence methods are used to control the beer brewing process. Beer quality can be predicted by applying FL rules to real process data, which helps in detecting higher alcohols, vicinal diketones, and fatty acids. Additionally, ANNs assist in monitoring and reporting the progress of beer fermentation. Beer is among the most ancient and widely consumed alcoholic drinks, appreciated by one-third of the global population. It possesses two main types of characteristics: sensory and visual. The visual aspects include color, clarity, turbidity, foam volume and retention, and overall appearance. On the sensory side, attributes such as aroma, bitterness, and mouthfeel are key [35]. Traditionally, evaluating these criteria requires significant manpower and time. However, using the CVS approach, this process can be automated. CVS inspects the beer’s visual qualities through digital photographs, utilizing components such as a camera, lighting, and a computer. It performs tasks like image capture, processing, and analysis to detect objects and extract qualitative data from samples. A crucial next step is digitization, where the image is converted into numerical data for further evaluation [35].

**Figure 9:**
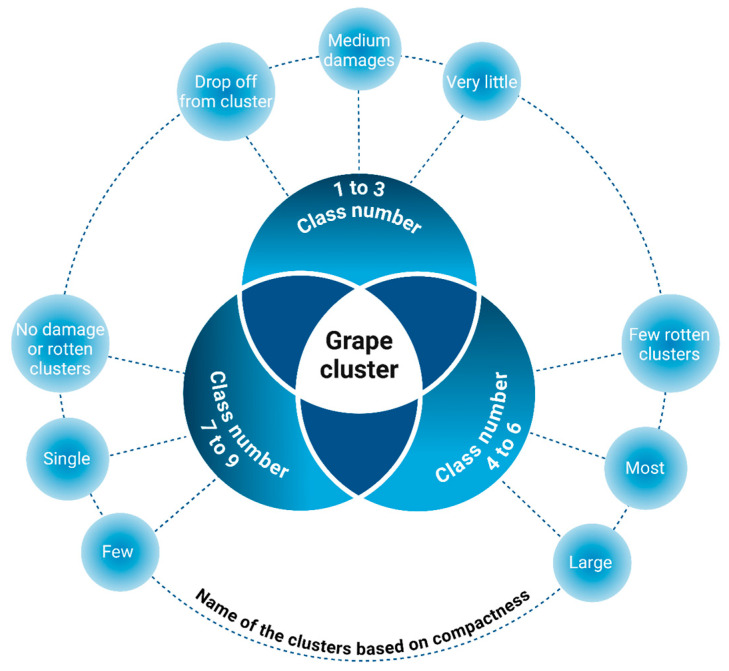
Grape cluster classification.

AI-powered mobile software for CV and image analysis is utilized to detect grape clusters that are unsuitable for wine production due to spoilage. This process is carried out in three essential steps. The initial step is image acquisition, where photographs are captured using mobile software in the vineyards one week prior to harvest. This allows for the evaluation of the grape clusters’ compactness. Expert panels can then manually rate the compactness based on guidelines provided by the International Organization of Vine and Wine (OIV 204) [119]. Cluster compactness is rated across nine categories, as shown in Figure 9. The second step is image processing, which serves as a precursor to the third step, segmentation. This segmentation step requires detailed data for each cluster to identify rotten or substandard clusters. Various methods and algorithms, such as K-means, Gaussian, and cross-validation, are employed to analyze and classify the clusters based on their quality [120].

#### 5.5.2. ANN in Non-Alcoholic Beverage

A specialized type of ANN, the Deep-CNN, is employed for the nutritional analysis of soft drinks to assist in managing weight gain and obesity. This approach estimates the nutritional content by evaluating factors such as bottle size and the ratio of the cap. This method is applicable to both carbonated and fruit-based beverages. By incorporating image processing within the CNN framework, the background is eliminated from the image, enabling precise calculation of the nutritional content [121].

#### 5.5.3. ANN in Hot Beverage

**Figure 10:**
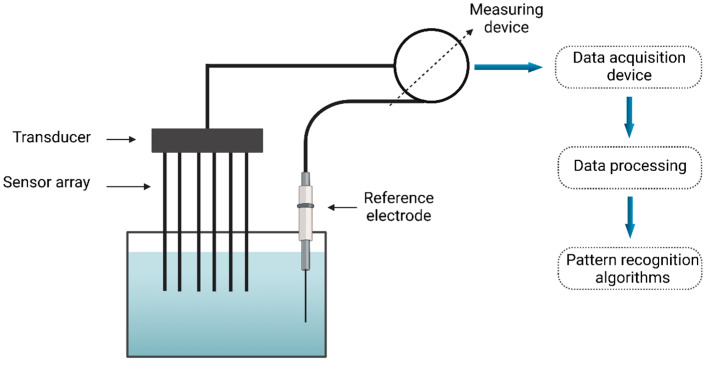
A schematic representation of e-tongue system.

The EN is a device developed to detect and evaluate odors, working similarly to the mammalian olfactory system by utilizing gas sensors [122,123]. It is extensively used in industries like wine and coffee for detecting scents. In the food and beverage industry, it helps monitor and ensure consistent product quality. The quality of roasted coffee is assessed through the coffee cupping method, in accordance with the standards established by the Technical Standards Committee of the Specialty Coffee Association of America [122]. First, 5 g of coffee beans are ground into a fine powder and steeped in boiling water for 3 to 5 min at a temperature range of 93–97 °C [124]. The cupping method is used to collect data, which are then predicted using a radial basis function ANN. Signals from the gas sensors in the EN are analyzed by a computer through LabVIEW software, with the data further processed using multivariable analysis techniques. The experiment is divided into three phases: first, examining the effect of temperature (ranging from 10 °C to 90 °C) on the smell characteristics of liquid coffee; second, categorizing the acidity levels of coffee based on various degrees of roasting; and finally, testing bitterness levels through human evaluation, guided by a radial basis function ANN [122]. Like the e-nose, the e-tongue (Figure 10) is also used to assess the quality of various beverages, including milk, coffee, tea, wine, and beer. The e-tongue detects key parameters such as saltiness, sourness, and bitterness [125]. The quality of tea can be evaluated using an e-tongue, which measures key flavor components such as theaflavin and thearubigin. Pu-erh tea samples of various grades and origins have been analyzed using the e-tongue along with other instrumental methods, including chemical analysis and electronic tongue technology [126]. A pulse voltametric e-tongue is used to determine the type of tea based on its age, which can be achieved through analysis using UV-VIS spectrophotometer-based equipment [127].

**Figure 11:**
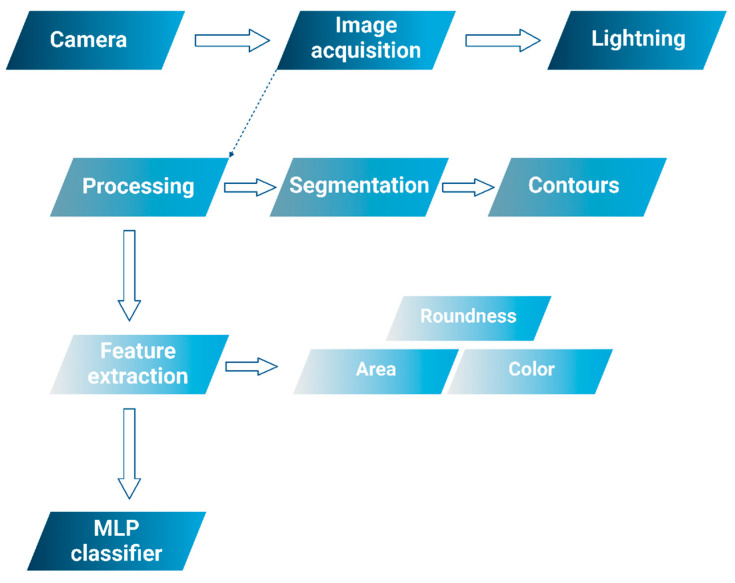
Defect detection using image processing algorithms.

The classification of coffee beans follows specific standards, taking into account category, defects, quality characteristics, and the nature of the resulting beverage [128]. Arabic espresso types are categorized based on type or defects, ranging from two to eight classifications. When assessing quality, it is important to consider factors that influence market perception and enhance the final product’s value. The classification process is part of the business evaluation, focusing on aspects such as shape, size, color, grain types, and beverage characteristics, all of which are integral to the marketing cycle (Figure 11). By utilizing image analysis, defects in the beans can be identified based on their size and shape, allowing for the removal of defective beans and ensuring the product’s quality is maintained [128].

### 5.6. AI in Bakery

Automation is advancing rapidly across industries, including the baking sector [129]. Bakery products come in a wide variety of shapes, sizes, and forms, making grading a crucial part of the production process, especially during handling and packaging where irregularities often occur [123]. In the past, quality assurance relied on human visual inspection. However, in modern bakery production, bread must undergo several processes to ensure consistency and quality. Bread making typically involves the introduction of live microorganisms, such as yeast, into the dough. If any step in the bread-making process is not executed correctly, the quality of the final product will be compromised [79]. In the bakery industry, a key focus is on optimizing production processes, utilizing resources efficiently, and implementing automatic control systems (ACSs) to improve product quality, lower costs, and boost profitability. The implementation of ACSs in bread-making enhances both productivity and efficiency, while also lowering electricity and fuel consumption during essential production processes. Additionally, optimizing resource efficiency and ensuring accurate information in bread production are crucial approaches for further improving operations [130].

Robotic technology systems in baking include automated control systems that monitor and regulate various critical factors. These sensors regulate critical parameters such as the rheological properties of dough and sourdough, the rising potential of the dough, the active acidity of the sourdough, the dough’s acidity and aroma, the dough’s shaping capabilities, the proofing time and temperature, humidity in the proofing chamber, and the dough’s weight [130]. The bread’s acidity, porosity, moisture content, dimensional stability, and core temperature, as well as the baking time, are implicitly controlled by a system of sensors along with a visualization interface. An intelligent decision-support system processes sensor data using components such as databases, knowledge bases, training modules, output blocks, and ESs, allowing for adjustments to the operational modes of ultrasonic systems for optimal bread production. This is accomplished by employing control mechanisms that enhance the management of effects across various technological environments. The ES for product quality control evaluates raw material parameters, enhancing the properties of dough, flour, and sourdough to improve the fortification qualities of the bread [79].

The entire baking process, from mixing to packaging, typically takes about three hours. Product quality assessment usually takes an additional hour, meaning the quality data cannot be immediately fed back to the mixer, oven, or other devices. Sensors designed for food quality play a crucial role by converting responses related to food properties into electrical signals, facilitating monitoring and control [131]. Sensors can operate in either online or offline modes. To assess bread quality, the inner portion of the loaf is typically examined using a camera after cutting. The quality of the loaf is assessed by measuring factors like dough porosity and size. These devices help monitor bakery products by delivering real-time data analysis according to the volume and variety of bread produced [79].

### 5.7. AI in Drying Fresh Foods

Advanced drying techniques that use physical fields, including microwave drying, radio frequency (RFQ) drying, infrared radiation (IR) drying, and ultrasonic drying, provide notable benefits for processing fruits and vegetables. These techniques can substantially reduce energy consumption and drying time, while also enhancing the drying process. Additionally, these methods help maintain the sensory attributes and nutritional value of fruits and vegetables, enhancing the overall quality of the dried products. Although physical field drying offers several benefits, it also poses challenges, including uneven drying, aroma loss, and nutrient degradation. However, these limitations can be mitigated by integrating AI into the drying process. AI-assisted physical field drying offers a promising solution for improving the drying of vegetables and fruits. Among the various physical field techniques, microwave drying stands out as a highly efficient and commonly applied method. By leveraging AI, the performance and outcomes of microwave drying can be further optimized. During the food drying and heat transfer process, the technique allows heat to penetrate materials and products without the need for an additional thermal gradient. This enables more efficient heat distribution throughout the product being dried [132]. Microwave technology has the potential to greatly improve both the drying rate and efficiency in the processing of vegetables and fruits. Research on potato chips has shown that microwave drying, particularly when combined with treatments using sodium chloride and sucrose, can substantially reduce the overall drying time [133]. Monton et al. [134] found that integrating convection drying with microwave drying significantly shortened the drying time of turmeric, while still meeting the quality standards set by the Thai Pharmacopoeia. Following the drying process, the moisture content was lowered to below 10.0% *v*/*w*, the volatile oil content surpassed 6.0% *v*/*w*, and curcumin levels exceeded 5.0% *w*/*w*. This combination of drying methods resulted in higher-quality dried products compared to using a single drying technique. Despite its advantages, microwave heating has certain drawbacks, such as uneven heating, shallow penetration depth, and the occurrence of phenomena like “expansion” [135]. To address these issues, Lv et al. [136] introduced an advanced intelligent microwave vacuum drying (MVD) system, incorporating low-field nuclear magnetic resonance technology to address these challenges. This device enables real-time monitoring of water content during the drying process of fruits and vegetables, offering more precise control and optimization. This system establishes a relationship between the water content of fruits and vegetables (M2) and the amplitude of the fitting signal (A2). The development of a linear model connecting M2 and A2 allows for accurate estimation of the drying endpoint in MVD, achieving a precision level greater than 95% (*p* > 0.950). It effectively monitors changes in water content during the microwave drying process. Additionally, Chen et al. [137] applied microwave drying to honeysuckle and introduced an FL control method for parameter regulation. This approach efficiently controlled the drying temperature, resulting in optimal drying outcomes. During the drying process, moisture content can be monitored using intelligent sensor technology, while the drying temperature can be regulated through the application of FL. This combination helps address the limitations of microwave drying, allowing for the determination of optimal drying parameters and improving product quality. Additionally, RFQ drying, a volumetric heating method, enables rapid and deep penetration of heat into food, further enhancing the drying process [138]. The capability of RFQ drying to penetrate food more thoroughly than microwave drying is attributed to its free-space wavelength, which is 20 to 360 times longer than that of microwaves. RFQ drying not only reduces drying time but also improves overall efficiency. To enhance drying speed, efficiency, and product quality, Zhou et al. [139] conducted a study using an RFQ vacuum drying system on kiwifruit slices, operating at a frequency of 27.12 MHz and a power level of 3 kW. The dried kiwifruit displayed vibrant color, high vitamin C content, and remarkable rehydration properties.

CVSs are effective tools for monitoring changes in shrinkage and color rate during the drying process of vegetables and fruits. By integrating a CVS into the RFQ drying process, issues related to color, nutritional content, and sensory quality can be effectively managed. Additionally, the application of control and analysis technologies, such as ANNs and FL, allows for the precise regulation of time and power, leading to improved drying outcomes.

IR radiation, characterized by its spectrum and directionality, is a type of electromagnetic radiation that falls within the wavelength range of 0.78–1000 μm [140]. IR drying offers several advantages, including rapid transient response, compact equipment, efficient convection and conduction, and significant energy savings, compared to microwave heating. As a result, it has become a key heat treatment technology in food processing, providing an effective and energy-efficient method for drying [140]. IR drying is beneficial for vegetable and fruit drying as it enhances the drying rate while helping to preserve their nutritional content. Adak et al. [141] investigated the influence of air temperature, drying conditions, and infrared power on the drying of strawberries. Their findings revealed that increasing infrared power reduced the drying time, while also improving the nutritional value of the strawberries, including higher antioxidant capacity and better retention of total anthocyanins and phenolic compounds. Although IR drying offers several advantages, its use in isolation can occasionally harm the sensory qualities of fruits and vegetables. Mihindukulasuriya et al. [142] observed that infrared drying of red pepper resulted in significant weight loss, a reduction in redness, and a decrease in capsaicin content. Such undesirable changes are not typically expected in the drying of vegetables and fruits. However, incorporating AI-assisted control within the drying process allows for more effective management of changes in color and volume, thus addressing these challenges. An intelligent fuzzy machine vision control system (FMCS) was developed by Nadian et al. [143], which combines FL and a CVS to regulate operational variables during infrared drying using mixed hot air. This system monitors the color and volume changes during kiwifruit drying and uses a genetic algorithm to optimize the quality of the dried products. The FMCS significantly reduces drying time compared to conventional hot air drying, cutting it from 40 min to 24 min (a 40% reduction). Additionally, it enhances product quality compared to standalone infrared drying, as evidenced by a reduction in color change from 7.9 to 2.1, a more than threefold improvement. The advancement of novel pretreatment and post-treatment technologies offers potential for significantly improving the quality of dried vegetables and fruits [144].

Ultrasound transmission through a medium generates various physical and chemical effects, making it a valuable tool in food drying applications [145]. Ultrasound, when applied to vegetables and fruits, can aid in preserving product quality after the drying process. Marcela et al. [146] found that using either an ultrasonic probe (direct method) or an ultrasonic bath (indirect method) for pretreating beet snacks significantly decreased color changes and cyanogen content, while also cutting the total processing time by approximately 26%. Wang et al. [147] investigated the effects of ultrasonic pretreatment on carrots before intermediate-wave IR drying. Their findings revealed that carrots subjected to ultrasound prior to IR drying preserved higher levels of β-carotene and demonstrated a superior rehydration ratio when compared to the untreated control samples. While ultrasonic treatment can enhance certain drying characteristics, it may also negatively affect the texture of vegetables and fruits. In their research, Rodriguez et al. [148] reported significant alterations in the microstructure of apples resulting from ultrasonic treatment during the drying process. However, by integrating AI technology to regulate ultrasonic power and control the rate of water loss during drying, these issues can be minimized. Additionally, Pu et al. [149] employed near-IR imaging to monitor the moisture distribution in mango slices during drying, which facilitated more uniform moisture removal and helped preserve the shape of the mango slices. In addition to addressing the limitations of various physical field drying methods for fruits and vegetables, AI technology can assist in optimizing the drying process. It provides valuable insights into the dynamics of physical field drying, enabling the development of more precise and accurate drying models. Taghinezhad et al. [150] conducted a study on the microwave drying of papaya, utilizing an ANN, particle swarm optimization, and the gray wolf optimization (GWO) algorithm to predict the relevant drying model. The GWO algorithm is a novel swarm intelligence optimization technique that emulates the leadership and hunting behaviors observed in gray wolves in their natural environment. This approach demonstrated strong analytical capabilities, accurately predicting the drying model of papaya. The results showed that this optimization method yielded the highest R^2^ value (0.9707) for the effective diffusion coefficient. Dai et al. [151] developed a combined IR convection (IRC) dryer that utilizes a support vector regression (SVR) algorithm, enhanced by an improved particle swarm optimization (IPSO) method. This system is capable of modeling and controlling the nonlinear behavior of drying particles. They proposed an IPSO-SVR model to manage and predict the drying process of grains using IRC, providing more accurate control over the drying dynamics. The development of these models enables better control and monitoring of the drying process, allowing for precise regulation of drying parameters. This not only helps reduce costs but also ensures the production of high-quality dried products. Table 2 provides an overview of other AI technologies applied to assist in the physical field drying of fruits and vegetables.

### 5.8. AI in Packaging and Sorting

In the food processing industry, the organization and packaging of food products are among the most labor-intensive and time-consuming tasks for manufacturing units. AI-based systems can effectively manage these processes, reducing the likelihood of errors and significantly enhancing the production efficiency of the industry. The development of AI-based systems for sorting and packaging in the food industry is challenging due to the variations in shape, color, and size of fruits and vegetables. To create an effective AI-driven system, a substantial amount of data is required to properly train the model, ensuring it performs the sorting and packaging tasks efficiently [167,168]. Numerous research teams have developed different systems to accomplish the same task. One such system, TOMRA, efficiently handles sorting with an impressive accuracy rate of 90%, leading to a significant increase in production speed. Currently, most sorting and packaging tasks in the industry are carried out by automated systems. The adoption of these technologies offers several benefits, including faster production rates, higher-quality output, and reduced labor costs. 

AI-based intelligent decision-making systems incorporate a range of tools and technologies, including high-resolution cameras, laser systems, X-ray imaging, and IR spectroscopy. These technologies enable a thorough analysis of various characteristics of food products, such as fruits and vegetables, at the input stage. In contrast to conventional systems that primarily assess products based on their external appearance, advanced systems like TOMRA have shown a 5–10% improvement in sorting and separation accuracy, specifically for potatoes [169,170]. A similar issue was addressed by a Japanese company that implemented a TensorFlow-based ML system, yielding impressive results and significant benefits for their production line. This system also demonstrated remarkable performance across various other food processing sectors. Additionally, each organization found that the AI-based system operated with greater precision. The success achieved in potato processing has prompted the expansion of AI technologies to other areas within the industry, with potential for further application across different departments of food processing.

## 6. Drawbacks and Limitation of AI

The development and deployment of different algorithms in AI offer unique advantages and disadvantages, often tailored to specific applications and industries. Understanding these benefits and limitations helps in selecting the most appropriate method for each context. Below is an analysis of several notable algorithms (Table 3).

Expert system:

ESs are AI programs designed to mimic human decision-making in specific domains. They provide high reliability and interpretability, allowing them to perform consistently and accurately in well-defined fields. For example, an ES can swiftly respond to input changes, adapting to new scenarios as data evolve. This quick responsiveness makes them suitable for time-sensitive applications such as diagnostics and resource management, where they often outperform human operators in terms of error rates. Furthermore, ESs can enhance the efficiency of resource use by optimizing processes based on predefined rules. However, implementing an ES involves significant initial costs due to the specialized design and the need for expertise in the specific domain. Additionally, ESs typically operate within a limited vocabulary and may struggle to communicate results effectively to non-specialists. This limited expressiveness may hinder broader application in fields that require adaptable or user-friendly interfaces.

Fuzzy logic:

FL systems are highly effective at handling uncertain or incomplete information, making them valuable in fields where data may lack precision, such as environmental monitoring or control systems. One advantage of FL is its simplicity and speed, allowing it to deliver quick results without requiring extensive computations. FL systems are also robust in noisy environments, maintaining performance despite data irregularities. Additionally, FL is cost-effective and offers flexibility, as rule expansion allows systems to evolve alongside changing requirements, enhancing the quality and safety of various applications. However, FL is restricted to specific rule sets, limiting its generalizability across different domains. These systems often rely on expert input to define comprehensive rules, which may hinder adaptability in complex scenarios where broader contextual understanding is necessary.

Artificial neural networks:

ANNs are well-suited for modeling complex, nonlinear relationships due to their ability to learn autonomously from data. They excel in pattern recognition tasks, such as image classification or NLP, as they can generalize across similar inputs. ANNs also perform well in noisy and fluctuating environments, as they are resilient to input variability. Furthermore, ANNs are cost-effective for nonlinear problem-solving, given their adaptability across diverse applications. Despite these advantages, ANNs are often criticized for their “black-box” nature, as their complex, multi-layered structure makes them difficult to interpret. Understanding how an ANN reaches a decision can be challenging, especially in critical applications that require transparency. Additionally, ANNs require significant training time and careful selection of layer configurations, as well as large, high-quality datasets to perform optimally, which can be resource intensive.

Convolutional neural networks:

CNNs are specialized for image-based tasks, making them highly effective for applications in object detection, image recognition, and classification. CNNs automatically learn crucial features within images, reducing the need for manual feature engineering and allowing the network to identify complex patterns across multiple layers of processing. This automated feature learning enhances CNN efficiency in visual tasks. However, CNNs demand high computational resources, making them costly and challenging to implement without advanced hardware. Additionally, CNNs are vulnerable to overfitting, especially when trained on small datasets, as they can become too finely tuned to specific data patterns. Their layered structure also complicates interpretation, making it difficult to understand how they reach decisions.

Computer vision systems:

CVSs offer a non-invasive method for assessing visual qualities like size, shape, and color without damaging samples, making them valuable for quality control in industries such as food production. A CVS enables automated visual inspection, which enhances efficiency and consistency in processes that require high accuracy. Additionally, a CVS can integrate with other AI methods to further boost accuracy, improving the precision of assessments. The primary drawbacks of CVSs are the high initial setup costs, as they require specialized equipment and software. Additionally, CVSs struggle in low-contrast environments, where optimal lighting is essential for reliable results. This dependency on controlled lighting conditions limits their applicability in environments with variable lighting.

In conclusion, each algorithm has distinct advantages that make it suitable for specific tasks and limitations that may restrict its broader application. Selecting the appropriate algorithm depends on the unique demands of the application, balancing efficiency, interpretability, and resource requirements.

## 7. Future Challenges

Significant advancements in food science have transformed food-based products into nutrient-rich supplements that help protect against various diseases. AI is increasingly being used to monitor changes in water quality and the impact of fertilizers on crop yields through the use of cameras and drones. In the production sector, AI plays a role in reducing food waste across industries and is used in restaurants to scan food items for their nutritional content. An important area of development involves methods for quantifying nanomaterials in food, with AI aiding intelligent packaging by detecting nanoscale substances in contact with food.

AI also bridges the gap between manufacturers by transferring vital information to the cloud, creating large datasets. Automated orchard harvesting, which enables fruit cultivation in previously unsuitable environments, helps save labor and optimize yields. AI is poised to address fluctuating supply and demand, narrow food hygiene standards, and improve supply chain management. Predicting the expiration dates of packaged food using sensors presents another challenge, but it could help consumers avoid foodborne illnesses by detecting spoilage in advance. Although the development of vendor applications is costly and mainly targeted at larger operations, expanding these AI-driven applications will facilitate the integration of restaurant robots in the near future.

## 8. Conclusions

The integration of AI across the agro-food supply chain represents a transformative force with the potential to address critical challenges facing modern agriculture and food systems. By leveraging state-of-the-art techniques such as ML, DL, FL, and ESs, AI enhances productivity, optimizes resource usage, and supports sustainable practices from farm to fork. Applications in agriculture—spanning preproduction, production, processing, and distribution—demonstrate AI’s ability to predict crop yields, improve irrigation efficiency, detect diseases, and enhance soil management, thereby advancing precision farming. Similarly, the food industry benefits from AI in areas such as quality control, safety monitoring, inventory management, and waste reduction. Innovations such as CVSs, e-noses, and advanced robotics streamline food processing, ensure safety, and mitigate resource wastage.

The adoption of AI-driven technologies extends to emerging domains like 3D food printing and intelligent packaging, underscoring AI’s potential to revolutionize the consumer experience and reduce environmental impact. Despite its transformative capabilities, challenges such as high implementation costs, the complexity of integrating AI with existing systems, and the need for comprehensive policies to ensure ethical and sustainable use remain significant barriers. Future developments in AI must prioritize inclusivity, accessibility, and cross-disciplinary collaboration to maximize their impact on global food security and sustainability.

In conclusion, AI is no longer a supplementary tool but a critical enabler of innovation in the agro-food system. Its strategic implementation across various stages of the food supply chain holds promise for creating resilient, efficient, and sustainable practices capable of feeding a growing global population while maintaining environmental balance.

## Figures and Tables

**Table 1:** The utilization of the CVS alongside AI in the food industry.

Application	Aims	AI Techniques	Findings	Reference
Tea	To differentiate between Iranian green tea and black tea.	-FL -DT	-The DT-based fuzzy systems can be effectively utilized for the automated and intelligent classification of Iranian black tea and green tea. -The REP-DT proved to be more advantageous than the J48 tree for creating a fuzzy classifier system.	[88]
Dry beans	To categorize various types of seeds based on their production.	-KNN -ANN -SVM -DT	-All the ML algorithms successfully classified the beans, with the SVM achieving the highest overall classification rate of 93.13%. This was followed by the DT, ANN, and KNN, which recorded classification rates of 92.52%, 91.73%, and 87.92%, respectively.	[89]
Barley flour	To forecast the characteristics of barley flour using the improved method.	-SVM -DT -KNN -RF	-The CVS model, enhanced with various learning algorithms, utilized the spatial pyramid partition ensemble method for classifying barley flour. This approach achieved accuracies of 75% with a KNN, 95% with SVM and an RF, and a perfect score of 100% with the DT method.	[90]
Bell pepper	To automatically assess the ripeness level of bell peppers.	-FL -ANN	-The RBF-ANN model demonstrates superior classification accuracy compared to FL, with maximum accuracies of 100% and 88% for the two models, respectively. -A system utilizing artificial vision was developed to predict the maturity of bell peppers by integrating a CVS with FL and an ANN.	[91]
Apple	For the purpose of differentiating between defective and normal apples.	-SVM -CNN	-The CNN integrated with the CVS model successfully classified apples with an impressive accuracy of 96.5%. This performance was significantly better than the traditional image processing method combined with the SVM classifier, which achieved an accuracy rate of only 87.1%.	[92]
Banana	For the purpose of categorizing bananas based on their ripeness.	-ANN -KNN -SVM -DT	-The ANN-based model system exhibits a superior classification rate relative to other algorithms, achieving an overall recognition rate of 97.75%.	[40]
Beer	To predict beer acceptability based on various sensory parameters.	-ML -ANN	-The integration of RoboBEER, CVS, and ANN algorithms facilitated the assessment of beer production based on customer acceptability and quality. -Seventeen ML algorithms were employed to identify the best-performing model, with results indicating that Bayesian regularization yielded the highest accuracy, achieving an R value of 0.92.	[93]
Lime	To estimate the weight of Indian lime fruits.	-ANFIS	-The system developed successfully estimated the weight of Indian sweet lime fruits with precision. -Various clustering methods were integrated with the ANFIS model to enhance accuracy in the classification system. The results indicated that FCM was the most effective in predicting the weight of sweet lime.	[94]
Mushrooms	To assess the quality of mushrooms based on their appearance.	-FL -ANN	-The ANN successfully estimated the weight of the mushrooms, while FL utilized data from the CVS to evaluate the quality of the mushrooms. -The image processing system achieved an accuracy of 95.6%.	[95]
Cape gooseberry	To categorize the ripeness of cape gooseberries.	-SVM -ANN -KNN -DT	-All models successfully classified the ripeness of cape gooseberries with high accuracy, achieving over 86% accuracy across different color spaces. This demonstrates that the system is an effective classifier.	[96]
Mango	To assess the mass of mangoes.	-ANN	-The system developed achieved a success rate of 97% and an efficiency coefficient of 0.99, utilizing either two or three input parameters.-The input parameters for the developed ANN model were derived from the data obtained from the CVS, enabling the model to successfully estimate the mass of the fruit.	
Rice	To manage the operation of rice whitening machines.	-FL	-The system’s flexible setup enables modifications to be made based on the preferences of individual rice mill operators. -The automatic control system that was developed demonstrated an average performance speed that was 31.3% greater than that of a typical human operator, and it also enhanced the quality of the output based on the decisions made by the system.	[97]
Pork loin	To evaluate the quality of pork loin in line with industry standards.	-SVM	-The model successfully predicted the quality of pork loin by analyzing its color and quality attributes in relation to industry demands.	[98]
Tomato	To determine the ripeness of fresh tomatoes.	-ANN	-Using the developed system, the ripeness of the tomato was detected with an accuracy of 99.31% and a standard deviation of 1.2%.	[99]
Passion fruits	To categorize passion fruits according to their ripeness levels.	-Multiclases SVM	-The developed system successfully classified the ripeness levels of passion fruits with an accuracy of 93.3% in just 0.94128 s.	[100]

Key: ANFIS, adaptive neuro-fuzzy inference system; ANN, artificial neural network; CVS, computer vision system; DT, decision tree; FCM, fuzzy C-means; FL, fuzzy logic; KNN, k-nearest neighbor; ML, machine learning; REP, reduced error pruning; RF, random forest; SVM, support vector machine.

**Table 2:** Advanced drying technology utilizing high-efficiency physical fields for AI applications.

Products	AI Technology	Drying Technology	Drying Condition	Reference
*Moringa olifera* leaves	ANN	Vacuum tray dryer	-Temperature: 40, 50, and 60 °C -Pressure: 0.8 bar -Dryer plates: 0.44 × 0.44 m Time: 30 min	[152]
Watermelon rind pomace	ANN	Solar air heaters	-Temperature: 40 °C -Energy consumption: 609 kWh.kg^−1^ and 318 kg CO_2_.kWh^−1^	[153]
*Stevia rebaudiana* Leaves	ANN	Microwave drying	-Power: 180 to 900 W	[154]
Green tea leaves	ANN	Fluidized bed drying	-Temperature: 50–80 °C -Air flow velocity: 7–9.5 m/s	[155]
Mushroom slices	ANN	Hot air impingement dryer	-Temperature: 55, 60, 65, 70, and 75 °C -Air velocity: 3, 6, 9, and 12 m/s -Sample thickness: 6, 9, and 12 mm	[156]
Walnut	ANN	Air-impingement technology	-Temperature: 40 °C, 45 °C, 50 °C, and 55 °C -Air velocities: 1, 2, 3, and 4 m/s -Moisture content: 10, 15, 20, and 25%	[157]
Potato slices	ANN	Conventional multi-stage convective cabinet dryer	-Air velocity: 3.58, 4.25, and 4.82 m/s -Temperature: 45 and 50 °C -Time: 240 min	[158]
Garlic slices	ANN	Osmo-sonicated dehydration	-Temperature: 20 °C -Time: 28.54 min -Osmotic concentration: 55.58% -Optimum responses: WL = 25.839%, SG = 3.557%, RR = 7.512, DR = 0.163 g H_2_O/g.dm/min, and AC = 15.229 mg/g	
Apple	ANN	Convective and microwave	-Temperature: 50, 60, and 70 °C -Air velocity: 1.0 m/s -Power: 90, 180, and 360 W	[159]
Mushroom	ANN	Microwave–hot air dryer	-Temperature: 23, 50, and 70 °C -Power density: 1.5, 2, and 2.5 W/g	[160]
Ginkgo biloba seeds	ANN	Microwave drying	-Power: 200, 280, 460, and 640 W	[161]
Onion	ANN	Custom designed fluidized bed dryer	-Temperature: 40, 50, and 60 °C -Air velocity: 2 and 3 m/s	[162]
Dragon fruit	ANN	Microwave vacuum drying	-Power: 200–600 W -Vacuum level: 3–9 kPa	
White Mulberry	ANN and FL	Infrared–convective drying	-Power: 500, 1000, and 1500 W -Temperature: 40, 55, and 70 °C -Inlet drying air speed: 0.4, 1, and 1.6 m/s	[163]
Mahaleb puree	ANN	Infrared drying	-Temperatures: 50, 60, 70, 80, and 90 °C	[164]
Apple slices	Real-time CVS	Intermittent microwave convective drying	-Power: 200–600 W -Temperature: 40 to 80 °C -Air velocities: 1–2 m/s -Pulse ratios: 2–6 -Drying rate: 0.014 to 0.000001 min^−1^ -Time: 27 to 244 min	[165]
Kiwifruit	CVS	Hybrid hot air–infrared drying	-Temperature: 70 °C -Thickness: 3 mm -Air velocity: 0.5 or 1.5 m/s	[166]

Key: ANN, artificial neural network; CVS, computer vision system; FL, fuzzy logic.

**Table 3:** Advantages and disadvantages of AI algorithms.

Algorithm	Advantages	Disadvantages
ES	-High reliability and interpretability. -Quick response to input changes. -Strong in practical tasks with low error rates. -Increases resource efficiency.	-High initial cost due to specialized requirements. -Limited vocabulary, posing communication challenges for non-experts.
FL	-Handles vague, incomplete data well. -Quick and simple results. -Tolerates noise, enhancing robustness. -Flexible rule expansion. -Cost-effective, improving quality and safety.	-Limited to specific rules, reducing generalization. -Often depends on expert input to create rules.
ANN	-Models complex functions accurately. -Resilient to noise and environmental changes. -Learns patterns autonomously. -Generalizes well. -Cost-effective and adaptable for nonlinear problems.	-Complex to interpret due to black-box nature. -Longer training time, requiring specific layer configurations. -Needs large, high-quality datasets.
CNN	-Highly effective for image-based tasks like object detection. -Identifies complex patterns across multiple layers. -Learns features automatically, reducing manual work.	-Computationally intensive, needing significant resources. -Prone to overfitting, especially with small datasets. -Difficult to interpret due to depth.
CVS	-Non-invasive, allowing visual assessments (size, shape, and color) without damaging samples. -Useful for automated visual inspection, especially in quality control. -Integrates easily with AI for accuracy.	-High setup costs for equipment and specialized software. -Limited in low-contrast environments, requiring optimal lighting.

## Data Availability

The data presented in this study are available in this article.
